# A Question of Sanatorium Control

**Published:** 1913-06-28

**Authors:** 


### The Editor's Letter-Box.

### "A Question of Sanatorium Control."

*To the Editor of* The Hospital.

Sir,—Referring to your Hospital and Institutional News of June 14, under the heading of "A Question of Sanatorium Control" you state "how is it possible for Dr. C. E. S. Fleming and others to introduce graduated labour until the medical officer has control of the outdoor servants?" With nearly twenty years' experience as a lay official of sanatoria and hospitals for consumption, I see no reason at all why it should not be quite simple to make the introduction of graduated labour under the present constitution of the Winsley Sanatorium. I know of several similar institutions where the secretary or other lay officer has the control of the outdoor servants and where graduated labour is in force.

It must be borne in mind that a successful secretary, superintendent, or steward of a sanatorium has an intimate knowledge of gardening, both utility and ornamental, and of all other work graduated labour patients are set to do, and consequently is well fitted to overlook such work. Doctors, it must be admitted, especially when young in years, have very little idea of growing vegetables and flowers or preparing the ground for same, to say nothing of tree felling and lopping, etc.

In a sanatorium that has at present no proper system, I would suggest that the medical officer writes out the grades, stating as near as possible the class of work for each grade and the hours per day each grade shall work. A copy of this should be deposited in the office of the secretary, together with, from time to time, the names of patients as they arrive at the graduated labour stage and advance from one stage to another. The lay official would then set the patients to work and arrange for the gardener or other skilled hand to give a lead where necessary, himself overlooking both patients and outdoor servants at the same time.

The patients' labour would then be of some value both to themselves and the institution, and it is common knowledge that patients are far happier when doing work that is useful than when put to tasks that have no value or meaning other than keeping them occupied.—I am, Sir, yours faithfully, A Sanatorium Steward.

June 17, 1913.

